# A PSTOL-like gene, *TaPSTOL*, controls a number of agronomically important traits in wheat

**DOI:** 10.1186/s12870-018-1331-4

**Published:** 2018-06-08

**Authors:** Matthew J. Milner, Rhian M. Howells, Melanie Craze, Sarah Bowden, Neil Graham, Emma J. Wallington

**Affiliations:** 1 The John Bingham Laboratory, NIAB, Huntingdon Road, Cambridge, CB3 0LE UK; 2 Plant and Crop Sciences Division, School of Biosciences, University of Nottingham, Sutton Bonington Campus, Loughborough, LE12 5RD UK

**Keywords:** Phosphate, PSTOL, Wheat, P, Seed size, Seed number, Flowering time, PUE

## Abstract

**Background:**

Phosphorus (P) is an essential macronutrient for plant growth, and is required in large quantities by elite varieties of crops to maintain yields. Approximately 70% of global cultivated land suffers from P deficiency, and it has recently been estimated that worldwide P resources will be exhausted by the end of this century, increasing the demand for crops more efficient in their P usage. A greater understanding of how plants are able to maintain yield with lower P inputs is, therefore, highly desirable to both breeders and farmers. Here, we clone the wheat (*Triticum aestivum* L.) homologue of the rice *PSTOL* gene (*OsPSTOL*), and characterize its role in phosphate nutrition plus other agronomically important traits.

**Results:**

*TaPSTOL* is a single copy gene located on the short arm of chromosome 5A, encoding a putative kinase protein, and shares a high level of sequence similarity to *OsPSTOL*. We re-sequenced *TaPSTOL* from 24 different wheat accessions and (3) three *T. durum* varieties. No sequence differences were detected in 26 of the accessions, whereas two indels were identified in the promoter region of one of the durum wheats. We characterised the expression of *TaPSTOL* under different P concentrations and demonstrated that the promoter was induced in root tips and hairs under P limiting conditions. Overexpression and RNAi silencing of *TaPSTOL* in transgenic wheat lines showed that there was a significant effect upon root biomass, flowering time independent of P treatment, tiller number and seed yield, correlating with the expression of TaPSTOL. However this did not increase PUE as elevated P concentration in the grain did not correspond to increased yields.

**Conclusions:**

Manipulation of *TaPSTOL* expression in wheat shows it is responsible for many of the previously described phenotypic advantages as *OsPSTOL* except yield. Furthermore, we show *TaPSTOL* contributes to additional agronomically important traits including flowering time and grain size. Analysis of *TaPSTOL* sequences from a broad selection of wheat varieties, encompassing 91% of the genetic diversity in UK bread wheat, showed that there is very little genetic variation in this gene, which would suggest that this locus may have been under high selection pressure.

**Electronic supplementary material:**

The online version of this article (10.1186/s12870-018-1331-4) contains supplementary material, which is available to authorized users.

## Background

Phosphorus (P) is an essential macronutrient that is required for all major developmental processes in plants and is considered to be one of the most limiting plant nutrients to global agricultural production. Approximately 70% of global cultivated land suffers from phosphate deficiency, making research into phosphate nutrition a priority for both scientists and farmers [1–3]. Phosphate is usually taken up in the orthophosphate forms (P_i_) which are available at low concentrations in the soil solution [4]. This low availability can be compounded by the soil chemistry in which soils high in clay can bind the P_i_ requiring large amounts of fertilizer to be applied to maintain high yields [4–6]. To compound the problem of P limiting growth and yield, recent estimates suggest that only 20–30% of the inorganic phosphate (P_i_) added to fields by farmers is assimilated by plants [7]. An increased understanding of how plants use P_i_ and the current inefficiency of P_i_ assimilation would be of economic benefit to farmers and also potentially alleviate environmental problems resulting from large scale agricultural production. Wheat (*Triticum aestivum L.*) is one of the most important crops worldwide and grown on more than 240 million hectares in 2016 [8]. P is one of the three most important nutrients for growth and yield improvement in wheat [9, 10]. Therefore an improvement in phosphate use efficiency (PUE) in wheat could potentially have a large global impact over 200 million hectares.

Recent advances in our understanding of the molecular mechanisms by which different plant species adapt to low-phosphate stress, the regulation and expression of phosphorus metabolism genes and alleles to deal with phosphate limitation, have enabled the design of more effective breeding strategies to produce highly phosphate efficient crops [11, 12]. A number of the underlying genes involved in the response of plants to low P are highly conserved and play similar roles in a number of diverse plant species including both model system and crop species. Thus, a greater understanding of the pathways involved in phosphate acquisition and signalling will allow breeders and plant molecular biologists to develop more efficient crops. Identification of these conserved pathways and genes from model organisms, and the subsequent transfer of this knowledge to crop species, would therefore allow farmers to optimize fertilizer use, resulting in increased food production efficiency with lower environmental cost.

One such locus which is believed to be important is the recent identification of the PUP1 locus from rice which contains a putative kinase gene called Phosphate Starvation Tolerance 1 (PSTOL) [13–16]. The PUP1 locus was originally identified in an upland variety of rice, Kasalath, yet is absent from most rice cultivars [17–19]. Rice varieties which have this genomic introgression containing the *PSTOL* gene, show increased biomass, increased root growth, increased tiller number and yield increases of up to 30% when grown under low P conditions whereas no deleterious consequences were seen when grown under normal soil fertility conditions [19, 20]. The identification of *OsPSTOL* and its role in helping rice tolerate low P conditions has led to the belief that we can engineer PUE into many crop species using translational science from gains in other model species.

Others have identified homologous *PSTOL*-like genes in both maize and sorghum, based upon QTL analysis and sequence homology. Further evidence of the role of PSTOL-like genes in PUE has been supported through QTL mapping rather than direct molecular characterization of candidate genes [21, 22]. Criteria to identify other potential PSTOL like genes has included identification of protein domains such as ATP kinase domains and on DNA sequence conservation meeting certain bioinformatic cut-offs for genes underlying these QTL. However some of these homologues appear to have differences in their gene structure such as number and length of introns and UTRs. Despite the lack of a highly conserved gene in most plant species, it is critical to understand whether other PSTOL genes exist in other important crop species and if they can be exploited.

Our study identifies a putative wheat PSTOL gene (*TaPSTOL*) and characterizes its role in PUE and other phenotypes of agronomic importance.

## Methods

### Gene identification

The *OsPSTOL* coding sequence from EMBL (AB458444.1) was used as a query for BLAST searches of the wheat genome (IWGSC 2014) and expressed sequence tag (EST) databases in ensembl, GenBank, Komugi and URGI [23–27]. Gene prediction was carried out using FGENESH [28]. Protein domain prediction was found using IntroProScan [29].

### Nullisomic (N) / Tetrasomic (T) wheat lines

PCR conditions for the verification of *TaPSTOL* homeologues were first optimized with wheat cv. Fielder DNA using a gradient from 53 to 63 °C T_m_ with and without 2% DMSO (final concentration) using FastStart™ Taq DNA Polymerase (SIGMA). All primers used in this study are listed in Additional file 1: Table S1. All three primer pairs amplified the predicted product under all conditions tested (Additional file 2: Figure S1). To reduce the stringency, the annealing temperature was then reduced to 55 °C to allow for small sequence variations and amplification of other distant homeologues using the nullisomic / tetrasomic wheat DNA [30].

### Creation of plasmid constructs

*TaPSTOL* was PCR amplified from genomic DNA extracted from wheat cultivar Chinese Spring using primers TaPUP-K46-F and TaPUP-K46-R with Phusion Hotstart II DNA Polymerase (Thermofisher). Amplicons were cloned into pJET2.1 (Thermofisher) and fully sequenced. The full length gene was then reamplified from pJET-TaPSTOL with TaPUP-K46-F-PvuII and TaPUP-K46-R-XbaI to add *Pvu*II and *Xba*I restriction sites plus a monocot ribosome binding site, CCACC [31], and cloned into pENTR-1A digested with *Dra*I and *Xba*I (Thermofisher). *TaPSTOL* was then recombined into the binary vector pSc4ActR1R2 [32] using a Gateway LR Clonase II Kit (Thermofisher) to create pRMH007. *TaPSTOL* was expressed *in planta* from the rice *Actin* promoter [33] and transcripts terminated by the *A. tumefaciens* nopaline synthase terminator (tNOS)*.* The first 350 bp of the *TaPSTOL* sequence selected to trigger silencing by RNAi, was amplified from the genomic sequence using the primers RNAi-F GW and RNAi-R GW, and recombined directly into pDONR221 using a Gateway BP Clonase II kit (Invtrogen). The pDONR *TaPSTOL* insert was subsequently recombined into the binary vector pACT-IR2 in a Gateway LR reaction to create pMM2, with the *TaPSTOL* RNAi hairpin cassette expressed from the rice *Actin* promoter *in planta*.

The 2.4 kb promoter region upstream of the *TaPSTOL* start codon was amplified from genomic DNA extracted from Chinese Spring using primers TaPUP-prom–F and TaPUP-prom-R with Phusion Hotstart II Polymerase (Thermofisher). The resultant amplicon was ligated into pCR-Blunt (Thermofisher) and sequenced. The promoter region was then reamplified with primers TaPUP-prom-13R and TaPUP-prom-SalI. The amplicon was digested with *Sal*I and ligated into pRMH013 digested with *Bmg*BI and *Sal*I. This intermediate vector containing the *TaPSTOL* promoter driving GUS and flanked by attL sites, was then recombined into pRLF12-R1R2-SCV to produce vector pRMH107. Additional file 3: Figure S2, shows the T-DNA regions from these constructs, which were transformed into wheat in this study.

Completed constructs were verified by restriction digest and sequencing before being electro-transformed into *Agrobacterium tumefaciens.* Plasmids were re-isolated from Agrobacterium cultures and verified by restriction digest prior to use in wheat experiments [34].

### Wheat transformation

Wheat cv. Fielder plants were grown in controlled environment chambers (Conviron) at 20 °C day/15 °C night with a 16 h day photoperiod (approximately 400 μE m^− 2^ s^− 1^). Immature seeds were harvested for transformation experiments at 14–20 days post-anthesis (dpa). Isolated immature wheat embryos were co-cultivated with *Agrobacterium tumefaciens* for 2 days in the dark [35]. Subsequent removal of the embryonic axis and tissue culture was performed as previously described [36]. Individual plantlets were hardened off following transfer to Jiffy-7 pellets (LBS Horticulture), potted up into 9 cm plant pots containing M2 compost plus 5 g/l slow release fertilizer (Osmocote Exact 15:9:9) and grown on to maturity and seed harvest in controlled environment chambers, as above.

### DNA analysis of transformed wheat plants

Plantlets which regenerated under G418 selection in tissue culture were transferred to Jiffy-7 pellets and validated using an *nptII* copy number assay relative to a single copy wheat gene amplicon, GaMyb, normalised to a known single copy wheat line. Primers and Taqman probes were used at a concentration of 10 μM in a 10 μl multiplex reaction using ABsolute Blue qPCR ROX mix (Thermofisher) with the standard run conditions for the ABI 7900 HT. The relative quantification, ΔΔCt, values were calculated to determine *nptII* copy number in the T_0_ and subsequent generations [37]. Homozygous and null transgenic lines were identified on the basis of *nptII* copy number and segregation analysis. WT Fielder plants were null segregates.

### Plant growth conditions

WT and transgenic lines were grown under low P conditions in sand and fertilized with nutrient solution [38] or under fertilized M2 compost conditions, as above. Total dry shoot weight, seed weight (yield per plant), seed number, seed size, tiller number and P concentration via ICP-MS were measured. Biological replicates each contained 14 plants per line and were grown until seed maturation. Tissues were allowed to dry for a further two weeks before harvesting. For low P conditions plants were germinated in sand and watered with 25 mL of a Magnavaca solution containing 3 μM KH_2_PO_4_, 1.3 mM NH_4_NO_3_, 3.52 mM Ca(NO_3_)_2_, 0.58 mM, KCl, 0.58 mM K_2_SO_4_, 0.56 mM KNO_3_, 0.86 mM Mg(NO_3_)_2_ 0.13 mM H_3_BO_3_, 5 μM MnCl_2_, 0.4 μM Na_2_MoO_4_, 10 μM ZnSO_4_, 0.3 μM CuSO_4_, Fe(NO_3_)_3_ and 2 mM MES (pH 5.5) twice a week until maturity. Plants were grown in a controlled growth chamber under 16 h light and 20 °C/15 °C day night temperatures.

### RNA expression analysis

Fielder seedlings were grown for seven days in 2.2 L pots containing Magnavaca solution as listed above and supplemented with either 1, 2.5, 5, 20 or 45 μM KH_2_PO_4._ Plants were grown for 7 days before harvesting tissue and separating the samples into root and shoot tissues for analysis. Total RNA was isolated from both roots and shoots for each P treatment using an RNeasy Kit (Qiagen) and treated with DNaseI (Thermofisher) prior to cDNA synthesis from 500 ng of total RNA using Omniscript RT Kit (Qiagen). The cDNA was diluted 1:2 with water and 0.5 μL was used as template in each RT-PCR reaction. Expression levels were quantified by quantitative PCR in triplicate reactions from three biological replications using SYBR Green JumpStartTaq ReadyMix (SIGMA) with the standard run conditions for the ABI 7900 HT. *TaPSTOL* expression was compared to two reference genes *TaUbiquitin* and *TaEF1α*. Primers used for amplification of transcripts were TaPSTOL-Q-F and TaPSTOL-Q-R, Ubi-F and Ubi-R for *Ubiquitin* [39] or EF1α-F and EF1α-R for *EF1α* [40]. Data shown is in comparison to *Ubiquitin* for ease as both reference genes showed similar differences in expression.

### Genomic comparison

Primers were designed to amplify a 3.3 kb fragment of *TaPSTOL* which included the 5′ upstream region and ORF from DNA extracted from Chinese Spring using the primers TaPUP-prom–F and TaPUP-prom-R. Genomic DNA was amplified from bread wheat varieties Alchemy, Banco, Bersee, Bridgadier Brompton, Claire, Copain, Cordiale, Fielder, Flamingo, Gladiator, Hereward, Holdfast, Kloka, Maris Fundin, Paragon, Rialto, Robigus, Slejpner, Soissons, Spark, Steadfast, Stetson, Xi19 and Chinese Spring, plus three wild *T. turgidum* ssp. *dicoccoides* accessions (PI 503314, PI 414722, and PI 428097) sourced from the USDA-ARS National Small Grains Collection. Genomic DNA was extracted using the Tanksley method [41].

### Gus staining of wheat tissues

Whole 10-day seedlings grown on sand watered once with hydroponic solution containing 3 μM P, were stained in X-Gluc solution (0.1 M NaPO_4_, 10 mM EDTA pH 7.0, 0.5 mM K Ferricyanide, 0.5 mM K Ferrocyanide, 1.0 mM X-Glucuronide, 0.1% Triton X100, pH 7.0) overnight at 37 °C, then destained in 70% ethanol [42].

### Digestion and elemental analysis of plant material

Dried samples of leaf, root and grain (~ 0.2 g) were digested and analysed for elemental content as described by Thomas et al. [43].

### Phosphate use efficiency calculations

Definitions of PPUE and PER were taken from [44]. PPUE was calculated as yield/ P concentration for an individual treatment. PER was calculated as yield / (P concentration * yield) for an individual treatment. ANOVAs were run using R and the aov and Tukey functions with the null hypothesis of no difference between lines. Tukey’s post hoc test was added to identify each significant interaction between the lines tested.

## Results

### Identification of *TaPSTOL*

Our *TaPSTOL* candidate was identified following BLAST searches [45] using the *OsPSTOL* coding sequence to the first wheat genome release (IWGSC 2014 and EST collections; this has subsequently been annotated as gene model Traes_5AS_AA3DC6A5F on ENSEMBL (IWGSC, 2014 release, Ensembl). The identified homologue is located on the short arm of chromosome 5A and shares 90% homology at the DNA level with the rice gene sequence. Further analysis showed that the rice and wheat predicted proteins share 74.2% identity and 92.7% similarity at the amino acid level (Additional file 4: Figure S3). *OsPSTOL* and *TaPSTOL* both possess single exons, and their predicted proteins are similar in size: 324 amino acids and 289 amino acids, respectively. InterProScan identified two protein domains which are predicted in both the wheat and rice *PSTOL* genes which include a protein kinase ATP-binding region signature (PS00107) located from amino acids 9–31 and a Serine/Threonine protein kinase active-site signature (PS00108) located from amino acids 126–138. The second best BLASTp hit to OsPSTOL in the wheat genome is encoded by a gene located on chromosome 3B, which shared only 54% amino acid identity suggesting that three sub-genome specific homologues were not present in wheat. A putative phosphate starvation activation domain thought to be bound by PHR1, also known as a P1BS binding site, was identified at − 1156 to − 1149 bp in the predicted promoter region of *TaPSTOL* [30]. To provide additional evidence that there is only a single gene present in wheat, the *TaPSTOL* sequence was also used to conduct a BLASTn search against *Triticum urartu* (A genome), *Aegilops speltoides* (B genome) and *Aegilops tauschii* (D genome) sequence databases. A single near perfect match was found for *T.urartu.* However no strong match was found in either the *A.speltoides* or the *A. tauschii* genome sequences. The best match for *A. speltoides* was 929 bp but with only 73% identity and for *A. tauschii* the best match was 266 bp with 85% identity [24, 27].

Due to the incomplete nature of the wheat genome assembly, we confirmed the absence of *TaPSTOL* homoeologues on chromosomes 5B and 5D by screening the Chinese Spring nulli-tetrasomic deletion lines [46]. PCRs using three primer combinations were designed using the 5A *TaPSTOL* promoter and coding region. This revealed only one copy of the PSTOL gene on chromosome 5A and no homoeologues on 5B or 5D (Additional file 2: Figure S1). To allow for potential small sequence variations in primer hybridisation sites, the stringency of primer annealing was reduced to 55 °C, but no additional amplicons were amplified. This suggests that only a single copy of *TaPSTOL* exists in Chinese Spring and that it is located on the short arm of chromosome 5A.

To search for sequence variation which might exist in the *TaPSTOL* gene or regulatory regions, we re-sequenced *TaPSTOL* from different wheat accessions: (1) the 22 founders of two bread wheat multiparent advanced generation inter-cross (MAGIC) populations (NIAB Elite MAGIC, [47]; NIAB Diverse MAGIC, [48]) that collectively capture 91% of genetic diversity in UK wheat; (2) spring varieties Fielder (USA) and Chinese Spring (China); and (3) three *T. durum* accessions (Additional file 5: Table S2). No sequence differences in any of the bread wheat lines tested were detected. A small variant was found in *T. durum* accession PI503314 which contained two indels in the promoter region. The first was a 13 bp insertion at − 355 and the second was a 34 bp deletion at − 704 bp. The A-genome sequence from other two dicoccoides accessions was identical to the hexaploid wheat sequence. No differences were found in the coding region in any of the lines tested.

### *TaPSTOL* gene expression

**Fig. 1:**
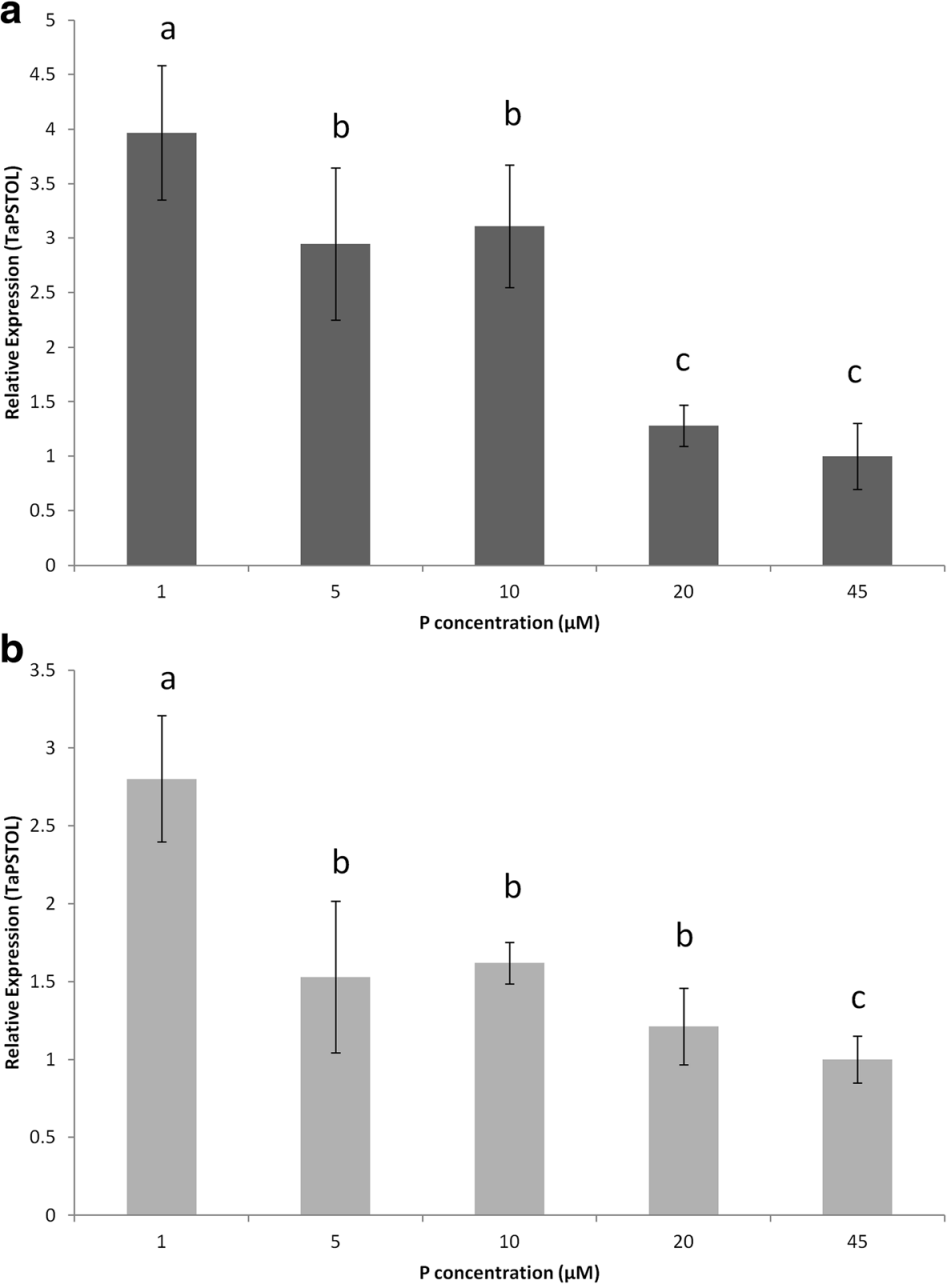
Expression of *TaPSTOL* mRNA in wheat grown under a range of P concentrations. Expression of *TaPSTOL* in (**a**) roots and (**b**) shoots is shown relative to *TaUbiquitin* mRNA after seven days growth in hydroponic solution. Error bars are SE of three biological replications. Letters represent a significant difference (*p* val < 0.05) between the same tissue type at any P concentration

**Fig. 2:**
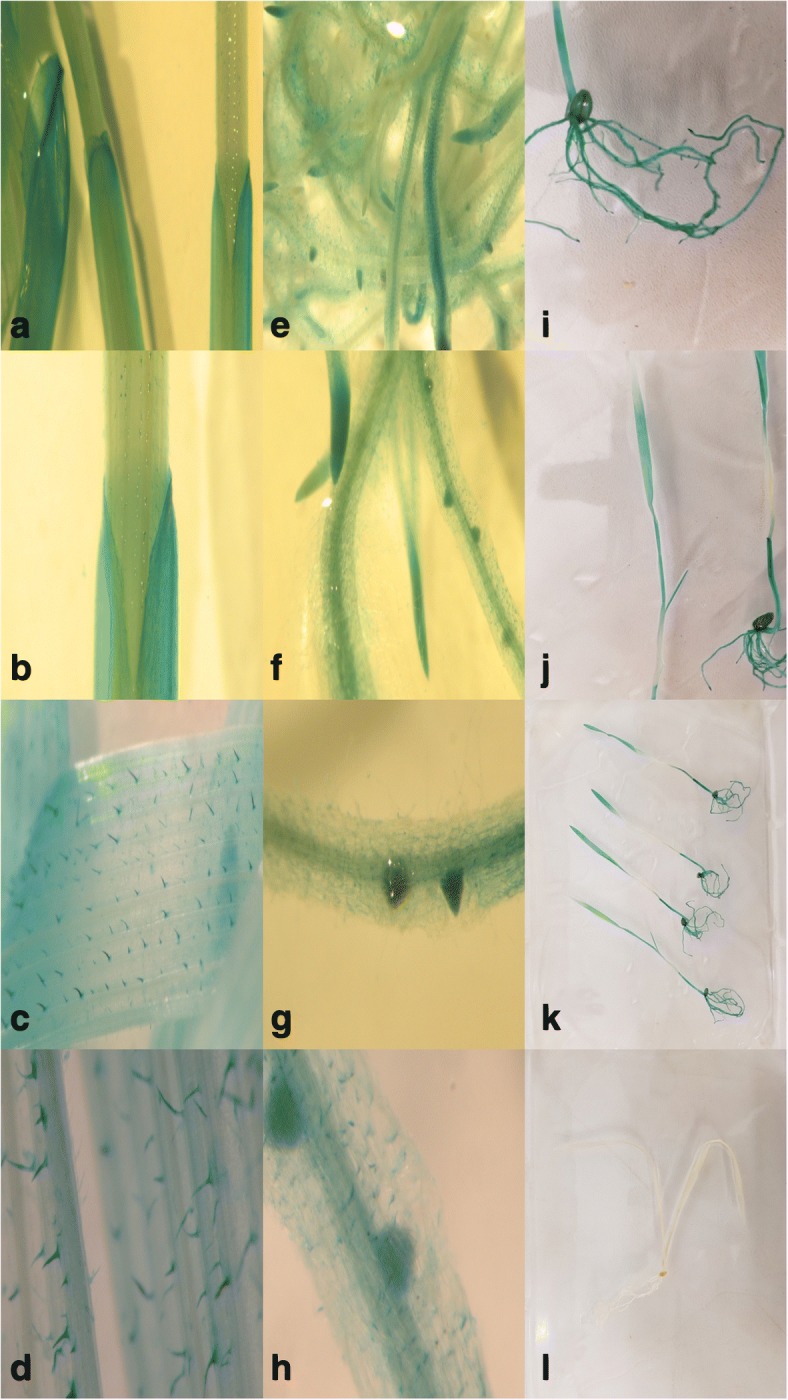
Characterisation of *TaPSTOL* promoter-*GUS* transcriptional fusions in wheat. Seedlings were grown in sand watered with –P hydroponic solution for 10 days after germination. Low magnification of **a** multiple coleoptiles, **b** a single coleoptile, **c** leaf trichomes. **d** Higher magnification of trichomes. Low magnification of (**e**) multiple roots, (**f**) isolated roots. Higher magnification of lateral root initials (**g**, **h**). Low magnification of root system (**i**), shoots (**j**), four plants showing *TaPSTOL:GUS* expression (**k**), non-transformed control plant (**l**) grown under same conditions

To further understand if Traes_5AS_AA3DC6A5F is the putative wheat *PSTOL* homologue, Fielder wheat plants were grown with varying levels of P (1, 5,10, 20 and 45 μM P) in hydroponics for seven days and expression of *TaPSTOL* was measured in both roots and shoots (Fig. 1). *TaPSTOL* is expressed relatively evenly in both root and shoot tissues, however *TaPSTOL* transcript abundance decreases with increasing P concentration in both tissue types. To further examine the expression of *TaPSTOL*, a 2.4 kb region directly upstream of the start codon was cloned and used to drive the expression of a *GUS* reporter gene. Characterization of 10 transformed wheat lines showed very low to no expression in roots, shoots or flowers of plants when grown under standard conditions in compost. When plants were grown under nutrient limiting conditions, *TaPSTOL* expression was found in the roots and shoots, with the highest expression in the root tips of both primary and lateral roots and coleoptiles (Fig. 2). Expression could also be seen in both the leaf trichomes and root hairs. Similar patterns of expression where observed in all ten lines tested.

### Characterization of *TaPSTOL* overexpression and RNAi knockdown lines in wheat

In order to evaluate the function of *TaPSTOL*, and compare to that of known OsPSTOL phenotypes, both over expression (OE) and RNA interference (RNAi) wheat transgenic lines were created. The expression level of *TaPSTOL* in thirty three OE and forty RNAi independent T_0_ lines was measured. Lines were selected for further study with a high over-expression (30× higher than wild type), low level overexpression (3X higher than wild type), a highly knocked down line (90% knock down) and a lower level of knock down (80% knock down), line here to referred as OE-1, OE-2, RNAi-1 and RNAi-2 respectively. Homozygous T_2_ plants were selected on the basis of *nptII* qPCR copy number analysis and resulting lines were phenotypically assessed under both low P conditions (3 μM P) in sand to better control P levels and standard P growth conditions, (M2 compost with 109 mg/l available P).

**Fig. 3:**
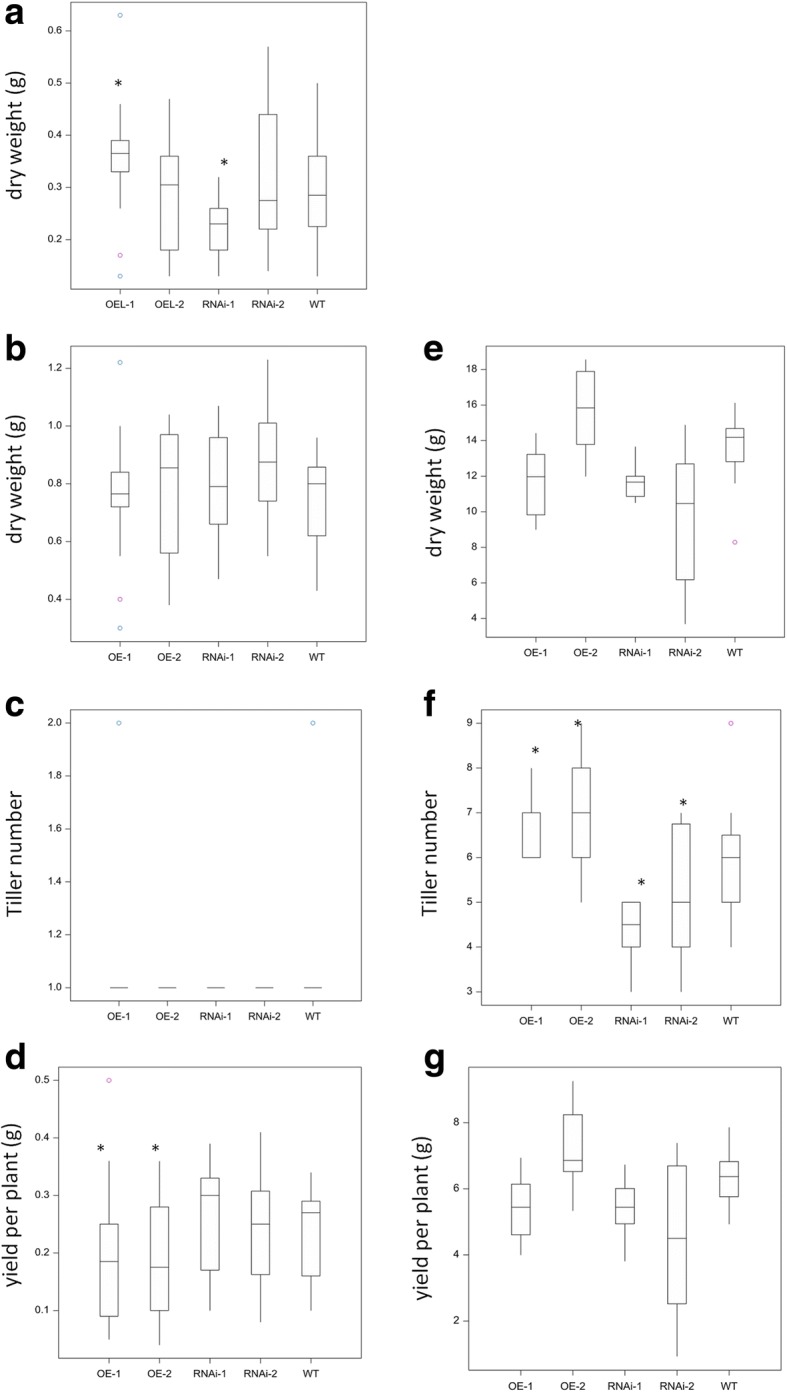
Agronomic measurements of OE and RNAi lines. Plants were grown under both low P (3 μM) conditions in sand to seed (**a**-**d**) or grown in M2 compost to seed (**e**-**g**). Dry weight of transgenic wheat **a** roots, or **b** shoots; **c** average tiller number; **d** yield per plant; **e** dry weight of shoots; **f**average tiller number; **g** yield per plant. Asterisk indicates significant difference *p* val < 0.05 to WT Fielder

For OE and RNAi lines grown on sand (fed twice a week with Magnavaca solution containing low P at 3 μM) a significant difference in the dry weight of roots from both the highest modified expression levels of OE and RNAi plants could be seen when grown to seed (ie OE-1 and RNAi-1, Fig. 3a). No significant differences in dry weight were found for the shoots of the OE or RNAi transgenic plants (Fig. 3b and e).

No difference in tiller number under low P conditions was seen in either the OE or RNAi lines (Fig. 3c). However, when grown on M2 compost, a significant difference in the tiller number was observed, with RNAi and OE lines showing a significantly lower and significantly higher tiller number, respectively (p val < 0.05) (Fig. 3f). Despite the difference in tiller number, modification of *TaPSTOL* expression showed no significant effect on total yield between the lines tested with growth in compost.

**Fig. 4:**
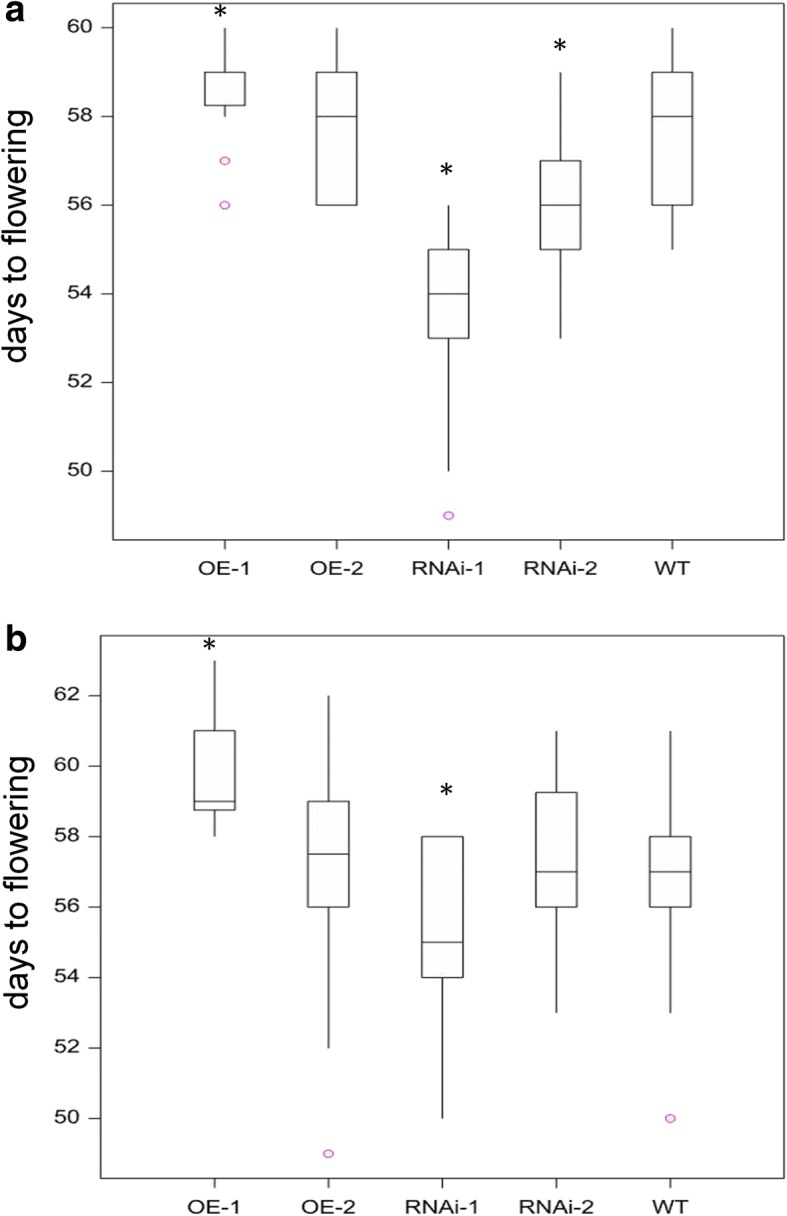
Flowering time measurements of OE and RNAi lines. Days to flowering, defined as Zadok stage 49, is shown under either **a** low phosphate conditions in sand (3 μM P) or **b** M2 compost conditions. Asterisk indicates significant difference *p* val < 0.05 to WT Fielder

During these experiments a novel phenotype was observed under low P conditions, namely a difference in the time to flowering, defined by time until growth stage 49 in days [49]. The RNAi lines were significantly earlier in flowering relative to the WT plants by almost two days (*p* val < 0.05)(Fig. 4a). As anticipated, the converse phenotype was seen for the OE lines as the time taken to flowering was significantly longer than the WT plants (p val. < 0.05). This difference in flowering time correlated with a significant negative effect on the total yield under low P conditions (3 μM) but not under M2 grown conditions for lines overexpressing *TaPSTOL* (Fig. 3d and g). The effect on flowering time was less pronounced under M2 compost growth conditions as only the highest overexpression line (OE-1) and highest knockdown line (RNAi-1) showed significant variation relative to WT Fielder (Fig. 4b).

A change in the overall root biomass production with a significant difference in root DW under low P for the both the highest (OE-1) and lowest (RNAi-1) *TaPSTOL* transgenic lines was observed (Fig. 3). While the overall total DW (root DW + shoot DW) under low P conditions was unaffected, there was an observable shift in DW from the root to shoot.

**Fig. 5:**
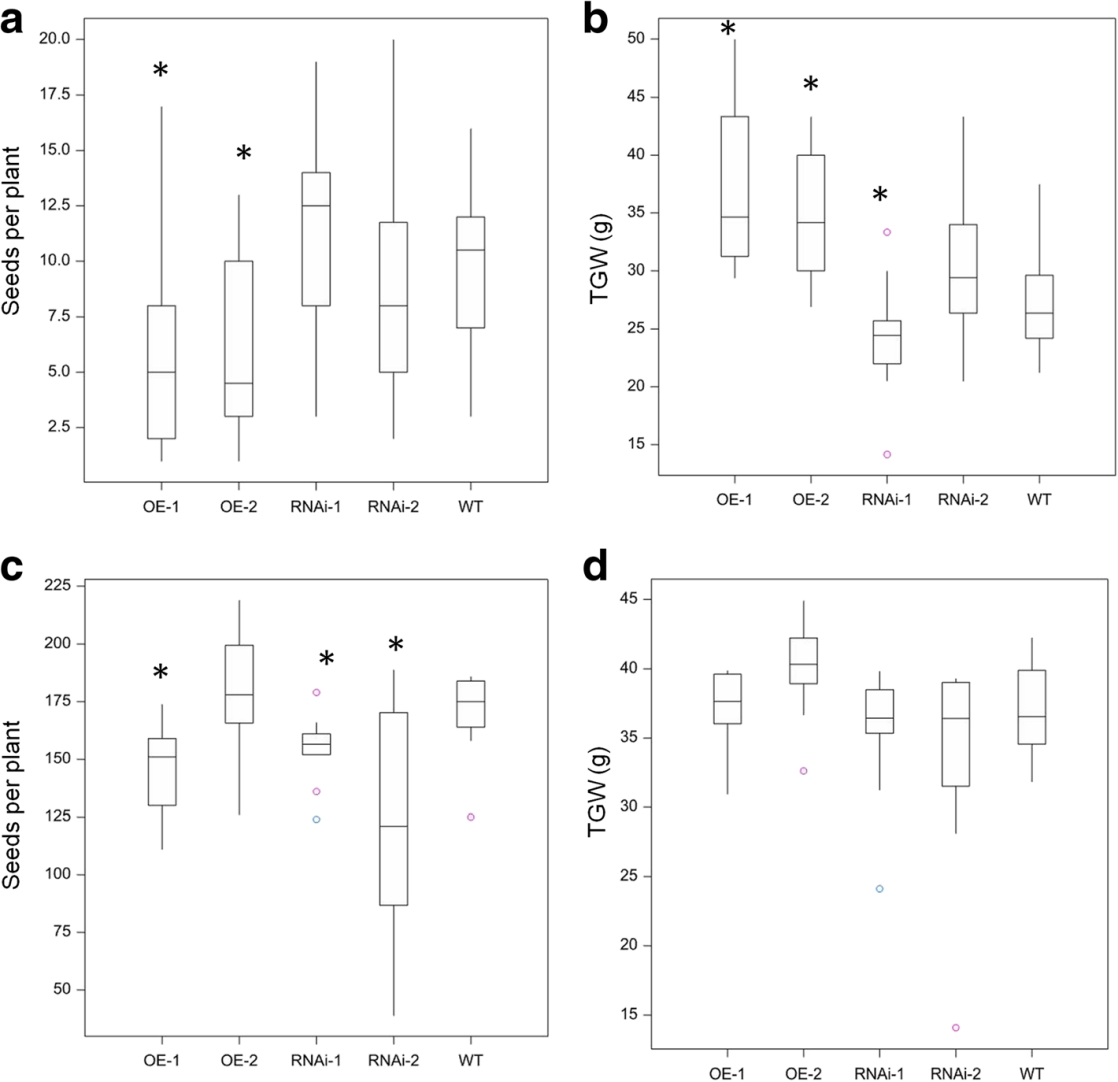
Seed yield parameters of OE and RNAi lines. Number of seeds per plant and thousand grain weight for *TaPSTOL* modified transgenic wheat plants when grown in **a** and **b** sand with low P conditions (3 μM); or **c** and **d** in M2 compost. Asterisk indicates significant difference, *p* val < 0.05 relative to WT Fielder

**Fig. 6:**
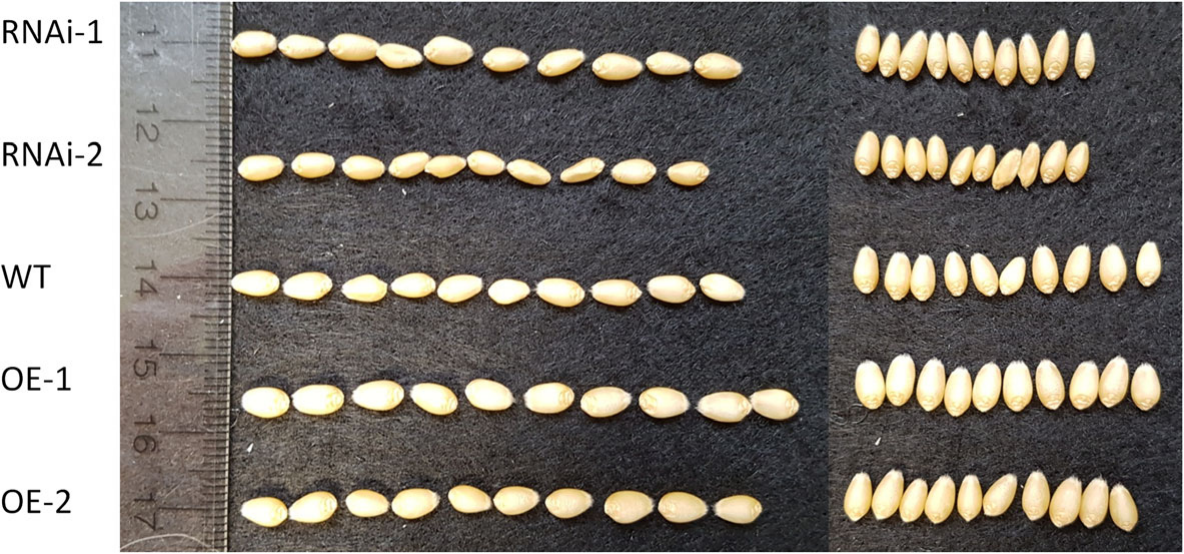
Comparison of seed size from *TaPSTOL* RNAi or OE plants. Ten seeds of *TaPSTOL* RNAi or OE lines plus WT Fielder grown on low P (3 μM P) on sand

*TaPSTOL* transgenic lines also showed differences in seed size. OE lines grown under P stress produced a decreased number of seeds compared to both RNAi and WT plants. In contrast, while OE lines produced far fewer seeds, each seed was 40–50% larger than the RNAi seeds and 30–40% larger than the WT seeds (Fig. 5b and d). This effect was mainly observed by an increase in the width of the seeds (Fig. 6) while the length remained similar.

**Fig. 7:**
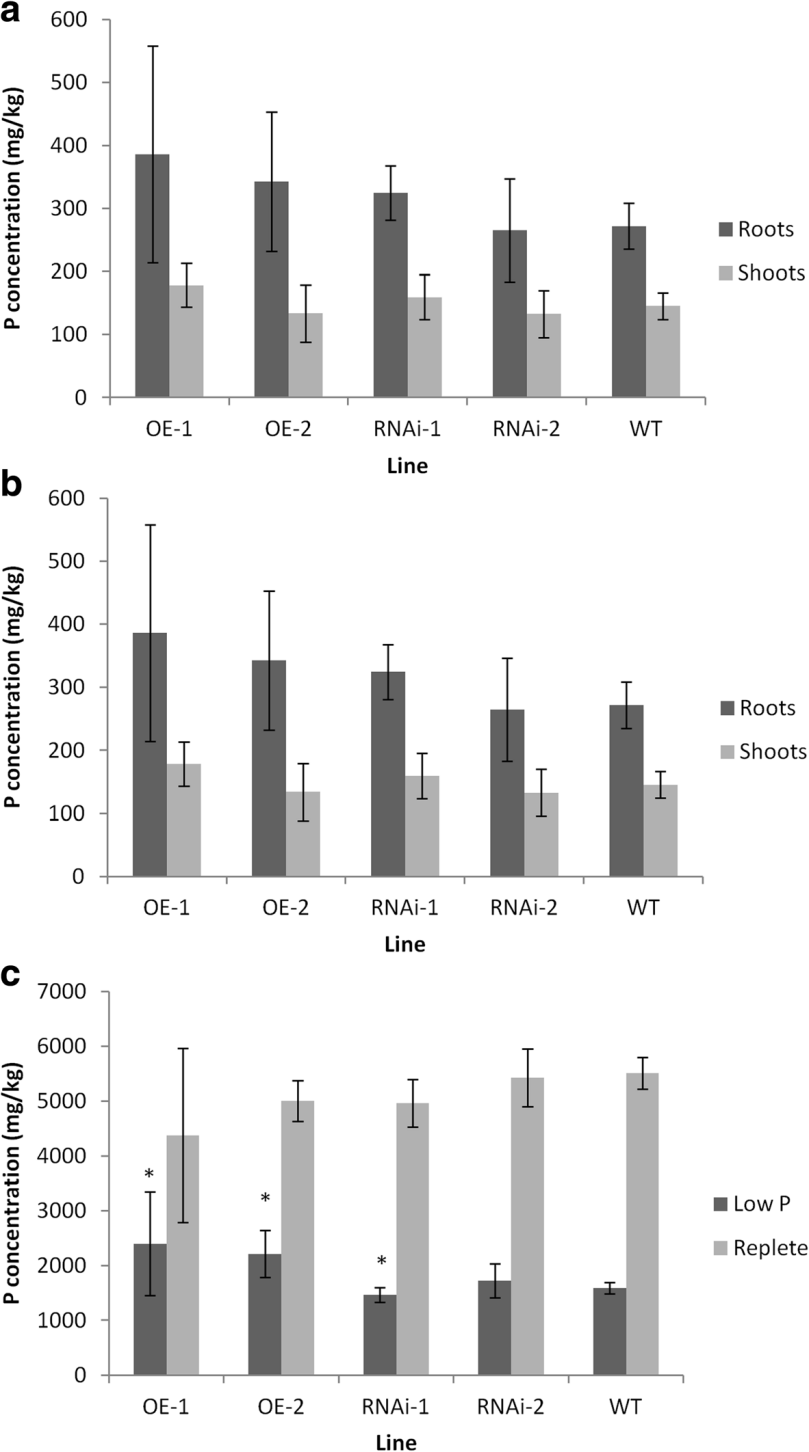
P concentration in roots, shoots and grains from *TaPSTOL* OE and RNAi plants. **a** Roots or shoots grown on low P in sand (3 μM); **b** Roots or shoots grown on M2 compost. **c** Grains harvested from *TaPSTOL* transgenic wheat plants grown on either low P or compost. Asterisk indicates significant difference, *p* val < 0.05 relative to WT Fielder

Total P concentration in the roots, shoot and seeds was also measured in five plants chosen randomly for each transgenic line grown under low P conditions and for shoots and seeds grown under compost conditions. No significant differences in P concentration were seen between root or shoot tissues grown under either condition in any of the sampled lines (Fig. 7a and b). There was a significant difference in the amount of P in the seeds of the RNAi-1 and OE-1 and OE-2 relative to WT (Fig. 7c). The differences in P concentration showed a lower P concentration in the RNAi line and higher P_i_ concentration in the OE lines (p val < 0.05). Other essential elements including K, Ca, Mg, Mn, Zn, Cu, Fe, Mo, S, and Co were also tested for significant differences between lines and no significant differences were found.

### The role of TaPSTOL in phosphorus use efficiency:

**Fig. 8:**
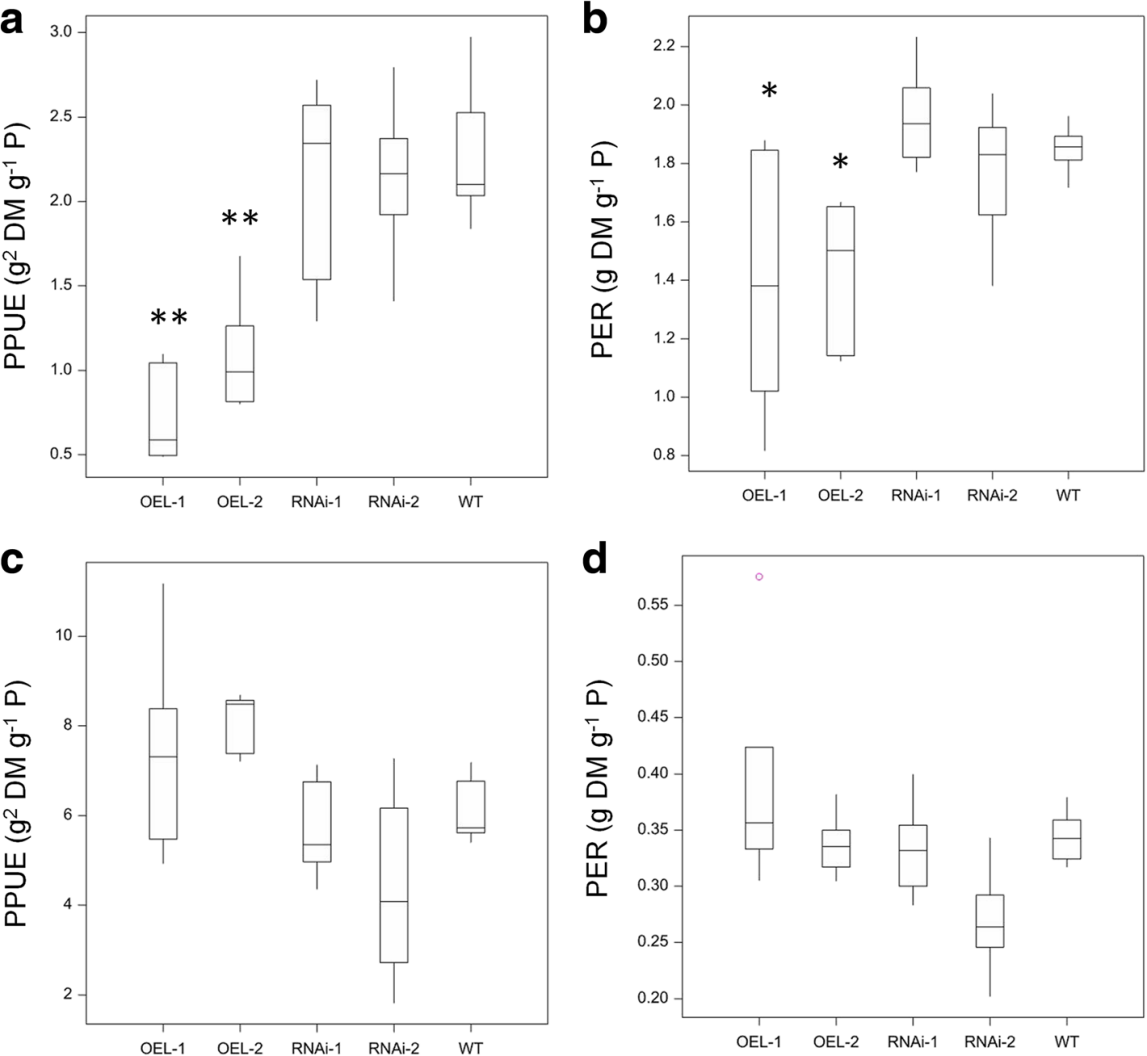
PPUE and PER of transgenic wheat plants. Plants were grown under both low P (3 μM) conditions in sand to seed (**a** and **b**) or grown in M2 compost to seed (**c** and **d**). Double asterisk indicates significant difference p val < 0.01 to WT Fielder

To understand how modification of *TaPSTOL* expression changes the PUE of wheat. The Physiological P use efficiency (PPUE) and the P efficiency ratio (PER) were measured (Fig. 8). There was a significant difference in the PPUE and PER when plants were grown on limiting P (*p* val < 0.001). The OE lines showed a significantly lower PPUE and PER, probably due to a significant increase in P in the grain accompanied by a decrease in yield. These differences in PPUE and PER were not observed under compost growth conditions, as no difference in the P concentration in the grain or differences in yield were seen.

## Discussion

The need to drive efficiency in global agricultural production has led to the elucidation of a number of key genes in breeding for phosphate efficient crops. Here we set out to identify and characterize the wheat homologue of *OsPSTOL*, which we denote as *TaPSTOL*, and compare the effect of manipulating its expression on a number of agronomic traits both under compost and low P conditions. The rice gene *OsPSTOL* has been shown to confer phenotypes beneficial to growth on P limiting soils, including increased grain yield, root and shoot biomass and increased tillering [14, 15, 18, 47]. Our data indicates that many of these PSTOL associated phenotypes are conserved between rice and wheat, including altered root growth under low P conditions (Fig. 3a), increased P content in the grain (Fig. 7c), and increased tiller number (Fig. 3f). There are, however, subtle differences as to when these phenotypes are exhibited in wheat as compared to rice; for instance, the increased tillering phenotype in wheat was only observed in plants grown in compost, whereas in rice, it was also found under P limiting conditions [14]. These differences in the conditions under which the increase in tillering occurs, might be a result of the stringent P limiting conditions in which the plants were grown in sand or the compost system used here to represent a replete P substrate. They could also be due to inherent differences between the two crop species, or the genetic background in which the *PSTOL* gene was studied in both rice and wheat. These differences are worthy of further study to identify the underlying mechanism of the *PSTOL* gene in both species and how *TaPSTOL* contributes to the regulation of tillering.

While many of the observed rice *OsPSTOL* mediated phenotypes were mirrored in wheat, further supporting the conserved nature of the *PSTOL* gene, changes in biomass and yield were not observed. This might be due the choice of germplasm used to test the effect of P efficiency in wheat or the P levels chosen might be too limiting or too generous to see the effects described in rice. There is currently no published data on P efficiency in the variety Fielder, but other work has suggested that natural variation in P efficiency does exist in wheat and this may alter the effect of TaPSTOL on P efficiency [10] within a specific variety. Recent findings in rice suggest that plants which contain the *PSTOL* gene are not the most efficient under low P conditions, which suggests that other genetic loci may play a greater role in increasing P efficiency [19]. Further work in both rice and wheat is required to further understand the processes by which P efficiency is increased, and the role played by *PSTOL* in these differences. While we found *PSTOL* mediated phenotypic effects in common between rice and wheat, our findings also highlight that the need for more understanding of how *PSTOL* genes, and their interactions with other genetic loci, may make crops more efficient under P limiting conditions.

We also identified two phenotypes associated with the *TaPSTOL* locus which were not observed in rice, showing that TaPSTOL modulates wheat flowering time and seed size. The timing of flowering is of great agronomic importance, as it entrains fertilization and seed set with favourable environmental conditions, thus helping farmers reach full yield potential in target agricultural environments [50]. Seed size is also an important component to driving yield gains, and we can now add another locus to those previously described as factors influencing grain size in wheat [51, 52]. It is interesting that the lack of genetic diversity at *TaPSTOL* in most of the wheat lines tested suggests that this gene may not have been found by traditional QTL mapping in most UK varieties, as no variation exists to be exploited in a mapping population. The lack of variation seen in the wheat cultivars tested, point to this region as having been under strong selection pressure. The majority of these bread wheat varieties originated from the UK, however additional varieties from Europe, USA and China were also included, yet did not show any variation at this locus. Only when the search is extended to the distantly related dicoccoides accessions, which originated from Israel, is some limited variation observed within *TaPSTOL*, limited to the promoter region. This finding is in contrast to *OsPSTOL* in rice where natural variation is found across species from a number of different continents in both the promoter and coding regions [17, 19]. The lack of DNA sequence variation might also be a consequence of a single *PSTOL* homoeologue present in the hexaploid wheat genome, located on chromosome 5A. Estimates for the number of genes present as just a single homoeologue in the hexaploid wheat genome are estimated to be in the range 3 to11% [53, 54].

Finally alteration of the expression of *TaPSTOL* led to changes in PUE as a decrease in yield and increase in P concentration when grown under P limiting conditions led to lower PUE scores for the over expression of *TaPSTOL* but not for the RNAi lines*.* As a positive increase in yield did not occur with the over expression of TaPSTOL to compensate for the increased P found in the grain, the PUE score was significantly lowered. This was not the case for the OsPSTOL where both an increase in yield along with an increase in P concentration of the tissues were found [18]. This difference in rice versus wheat might be due to some other part of the signalling cascade needing to be identified and combined with increased *PSTOL* expression to help drive the yield increases under limiting P growth conditions.

## Conclusions

We have identified and characterized the *PSTOL* gene in wheat and demonstrated that it controls a number of agronomic characteristics important to breeders and farmers, including altering root growth under P deficiency conditions and increased P content in the grain. While some of these phenotypes are conserved with *PSTOL* genes in other plant species, other phenotypes manifest themselves under slightly different P conditions. In addition, we show that while *PSTOL* may not influence yield as it does in rice, *TaPSTOL* modulates flowering time and seed size in wheat. These results demonstrate *PSTOL* genes in multiple crop species may be important targets for improving agronomic performance, both under P limiting and non P limiting conditions.

## Additional files

Additional file 1: **Table S1.** Primers used in this study. (DOCX 33 kb)

Additional file 2: **Figure S1.** Location of gene-specific primers and PCR amplification of *TaPSTOL* genomic regions on DNA extracted from nullisomic (N) / tetrasomic (T) wheat lines. The PCR amplicons correspond to a promoter region (PCR1, 1458 bp), a promoter and coding region (PCR2, 553 bp), and a coding region (PCR3, 870 bp), to demonstrate that *TaPSTOL* is only present on chromosome 5A. (DOCX 63 kb)

Additional file 3: **Figure S2.** T-DNA structure for the three *TaPSTOL* constructs used in this study. (DOCX 30 kb) (DOCX 80 kb)

Additional file 4: **Figure S3.** ClustalW alignment of the rice and wheat PSTOL predicted proteins. Conserved amino acids are coloured in red. (DOCX 162 kb)

Additional file 5: **Table S2.** Geographical origin of wheat accessions used in this study. (DOCX 30 kb)

## Abbreviations

dpa: Days post anthesis
DW: Dry weight
EST: Expressed sequence tag
ICP-MS: Inductively coupled plasma-mass spectrometry
MAGIC: Multiparent advanced generation inter-cross
N: Nullisomic
OE: Over expression
P: Phosphorus
PER: P efficiency ratio
P_i_: Inorganic phosphate
PPUE: Physiological P use efficiency
PSTOL: Phosphate starvation tolerance
PUE: Phosphate use efficiency
RNAi: RNA interference
T: Tetrasomic
tNOS: Nopaline synthase terminator
