# Supplementary material for: A PSTOL-like gene, TaPSTOL, controls a number of agronomically important traits in wheat
Source: BMC Plant Biol. 2018 Jun 8;18:115. doi: 10.1186/s12870-018-1331-4 (PMC5994007; doi:10.1186/s12870-018-1331-4)
Supplement: Supplementary file 1 — Table S1. Primers used in this study. (DOCX 33 kb) [file 12870_2018_1331_MOESM1_ESM.docx]

**Additional File 1: Table S1.** Primers used in this study.

| **PSTOL Primers** | **Sequence 5’-3’** | **Use** | **Target gene/ gene prediction model** |
| --- | --- | --- | --- |
| TaPUP-K46-F | ATGACCAGCTCTTTTGCTCACAAGC | CDS PCR | Traes_5AS_AA3DC6A5F |
| TaPUP-K46-R | TCAACATGCTTTTGGTGGAAGAC | CDS PCR | Traes_5AS_AA3DC6A5F |
| TaPUP-K46-F-PvuII | CTAGCTCAGCTGCCACCATGACCAGCTCTTTTGCT | CDS PCR | Traes_5AS_AA3DC6A5F |
| TaPUP-K46-R-XbaI | CATCTAGATCAACATGCTTTTGGTGG | CDS PCR | Traes_5AS_AA3DC6A5F |
| RNAi-F GW | GGGGACAAGTTTGTACAAAAAAGCAGGCTATGACCAGCTCTTTTGCTCACAAGC | RNAi region PCR | Traes_5AS_AA3DC6A5F |
| RNAI-R GW | GGGGACCACTTTGTACAAGAAAGCTGGGTTACTCGAGGCCTCGAGCAAT | RNAi region PCR | Traes_5AS_AA3DC6A5F |
| TaPUP-prom–F | ACCGGATACAGTGCTAAGTTA | Promoter PCR | Traes_5AS_AA3DC6A5F |
| TaPUP-prom-R | CCTTCTCACTTCCGGATAAG | Promoter PCR | Traes_5AS_AA3DC6A5F |
| TaPUP-prom-seq-2R | CGTCTGATGATGAAACTAATGAT | Promoter | Traes_5AS_AA3DC6A5F |
| TaPUP-prom-13R | AAGATATACTTATCCGGAAGTGAGAAGGATG | Promoter-GUS PCR | Traes_5AS_AA3DC6A5F |
| TaPUP-prom-SalI | ATTCAGTCGACACCGGATACAGTGCTAAGTTACAGG | Promoter-GUS PCR | Traes_5AS_AA3DC6A5F |
| TaPSTOL-Q-F | ATGACCAGCTCTTTTGCTCACAAGC | Expression | Traes_5AS_AA3DC6A5F |
| TaPSTOL-Q-R | TACTCGAGGCCTCGAGCAAT | Expression | Traes_5AS_AA3DC6A5F |
| MM53 | TACTCGAGGCCTCGAGCAAT | nullisomic (N) / tetrasomic (T) wheat lines | Traes_5AS_AA3DC6A5F |
| MM475 | GTGCTGTCTTTTCAGATGTAATAGTCG | nullisomic (N) / tetrasomic (T) wheat lines | Traes_5AS_AA3DC6A5F |
| Ubi-F | CCTTCACTTGGTTCTCCGTCT | Expression | TRIAE_CS42_7AL_TGACv1_558035_AA1789320; TRIAE_CS42_7BL_TGACv1_578170_AA1890500; TRIAE_CS42_7DL_TGACv1_605239_AA2005480 |
| Ubi-R | AACGACCAGGACGACAGACACA | Expression | TRIAE_CS42_7AL_TGACv1_558035_AA1789320; TRIAE_CS42_7BL_TGACv1_578170_AA1890500; TRIAE_CS42_7DL_TGACv1_605239_AA2005480 |
| EF1α-F | TGGTGTCATCAAGCCTGGTATGGT | Expression | TRIAE_CS42_U_TGACv1_641551_AA2097790; TRIAE_CS42_4BL_TGACv1_320923_AA1051800; TRIAE_CS42_4DL_TGACv1_342857_AA1124090 |
| EF1α-R | ACTCATGGTGCATCTCAACGGACT | Expression | TRIAE_CS42_U_TGACv1_641551_AA2097790; TRIAE_CS42_4BL_TGACv1_320923_AA1051800; TRIAE_CS42_4DL_TGACv1_342857_AA1124090 |
| GamyB1F | GATCCGAATAGCTGGCTCAAGTAT | Copy number QPCR | *GaMYB* |
| GamyB2R | GGAGACTGCAGGTAGGGATCAAC | Copy number QPCR | *GaMYB* |
| GamyB1P | [Joe]CGTGGCTCCTGCGATGCAGC[TAMRA] | Copy number QPCR | *GaMYB* |
| Npt2B2F | CTCCTGCCGAGAAAGTATCCA | Copy number QPCR | *npt*II |
| Npt2B4R | GCCGGATCAAGCGTATGC | Copy number QPCR | *npt*II |
| Npt2B2P | [FAM]TGGCTGATGCAATGCGGCG[TAMRA] | Copy number QPCR | *npt*II |
