# Supplementary figures and images for: A PSTOL-like gene, TaPSTOL, controls a number of agronomically important traits in wheat
Source: BMC Plant Biol. 2018 Jun 8;18:115. doi: 10.1186/s12870-018-1331-4 (PMC5994007; doi:10.1186/s12870-018-1331-4)

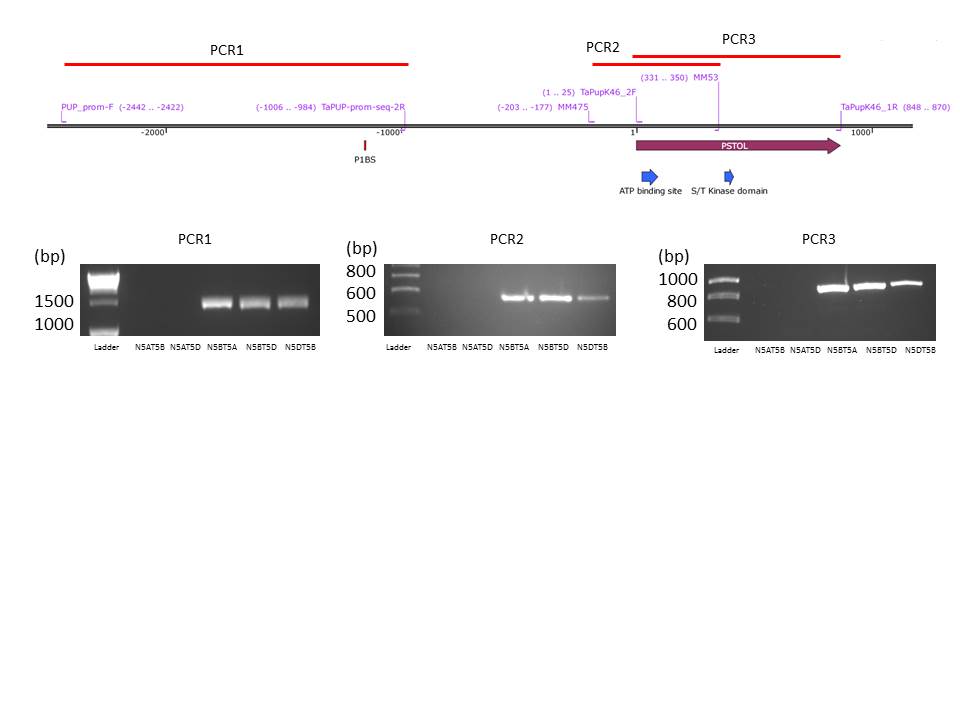

Supplement: Supplementary file 2 — Figure S1. Location of gene-specific primers and PCR amplification of TaPSTOL genomic regions on DNA extracted from nullisomic (N) / tetrasomic (T) wheat lines. The PCR amplicons correspond to a promoter region (PCR1, 1458 bp), a promoter and coding region (PCR2, 553 bp), and a coding region (PCR3, 870 bp), to demonstrate that TaPSTOL is only present on chromosome 5A. (DOCX 63 kb) [file 12870_2018_1331_MOESM2_ESM.docx]

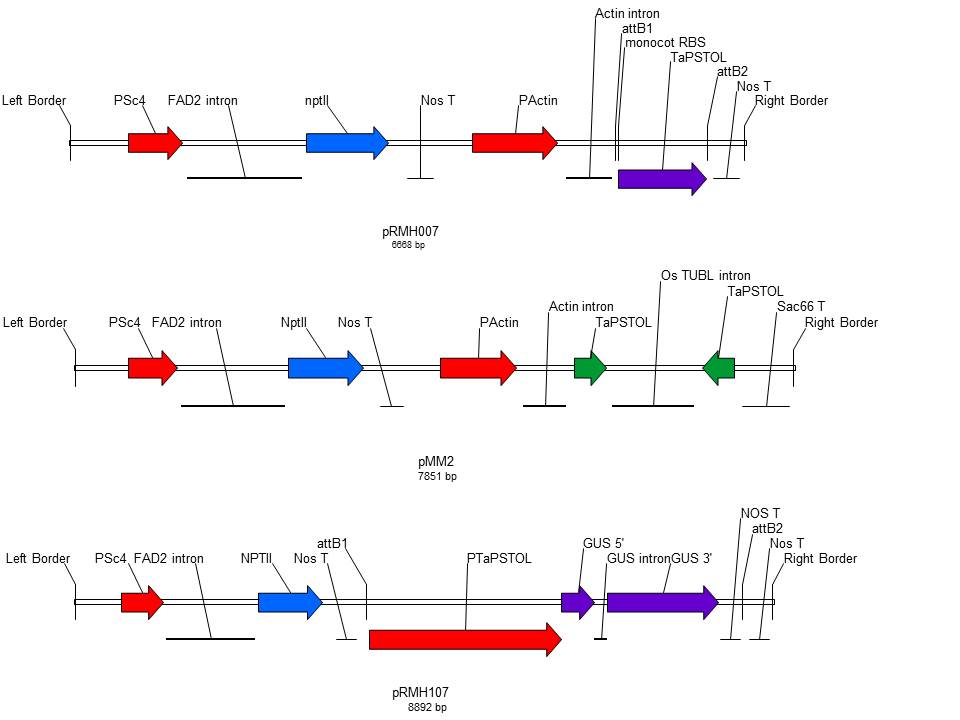

Supplement: Supplementary file 3 — Figure S2. T-DNA structure for the three TaPSTOL constructs used in this study. (DOCX 30 kb) (DOCX 80 kb) [file 12870_2018_1331_MOESM3_ESM.docx]

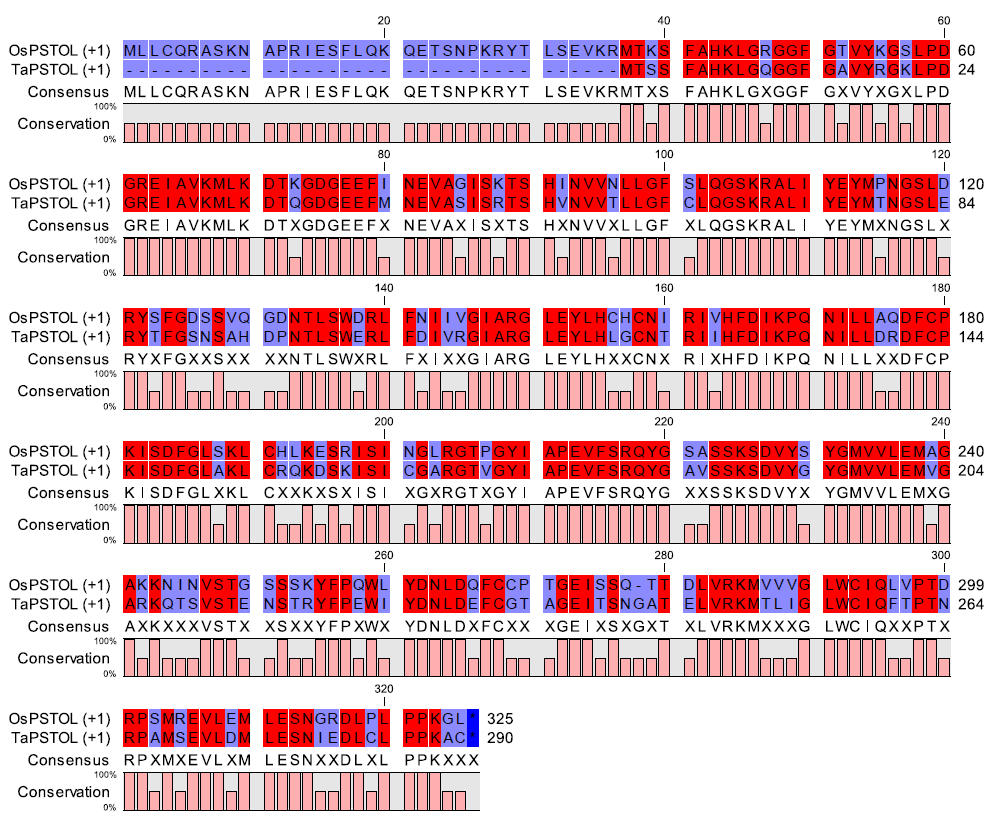

Supplement: Supplementary file 4 — Figure S3. ClustalW alignment of the rice and wheat PSTOL predicted proteins. Conserved amino acids are coloured in red. (DOCX 162 kb) [file 12870_2018_1331_MOESM4_ESM.docx]
