# Supplementary material for: A PSTOL-like gene, TaPSTOL, controls a number of agronomically important traits in wheat
Source: BMC Plant Biol. 2018 Jun 8;18:115. doi: 10.1186/s12870-018-1331-4 (PMC5994007; doi:10.1186/s12870-018-1331-4)
Supplement: Supplementary file 5 — Table S2. Geographical origin of wheat accessions used in this study. (DOCX 30 kb) [file 12870_2018_1331_MOESM5_ESM.docx]

**Additional File 2**: **Table S2.** Geographical origin of wheat accessions used in this study.

| **Species** | **Variety or accession number** | **Origin** |
| --- | --- | --- |
| *T.aestivum* | Alchemy | UK |
| *T.aestivum* | Banco | Sweden |
| *T.aestivum* | Bersee | UK/France |
| *T.aestivum* | Bridgadier | UK |
| *T.aestivum* | Brompton | UK |
| *T.aestivum* | Claire | UK |
| *T.aestivum* | Copain | France |
| *T.aestivum* | Cordiale | UK |
| *T.aestivum* | Fielder | USA |
| *T.aestivum* | Flamingo | Netherlands/Denmark |
| *T.aestivum* | Gladiator | UK |
| *T.aestivum* | Hereward | UK |
| *T.aestivum* | Holdfast | UK |
| *T.aestivum* | Kloka | Germany/UK/Denmark |
| *T.aestivum* | Maris Fundin | UK |
| *T.aestivum* | Paragon | UK |
| *T.aestivum* | Rialto | UK |
| *T.aestivum* | Robigus | UK |
| *T.aestivum* | Slejpner | Sweden/Denmark |
| *T.aestivum* | Soissons | France |
| *T.aestivum* | Spark | UK |
| *T.aestivum* | Steadfast | UK |
| *T.aestivum* | Stetson | UK |
| *T.aestivum* | Xi19 | UK |
| *T.aestivum* | Chinese Spring | China |
| *T. turgidum* ssp. *dicoccoides* | PI 503314 | Israel |
| *T. turgidum* ssp. *dicoccoides* | PI 414722 | Israel |
| *T. turgidum* ssp. *dicoccoides* | PI 428097 | Israel |
